# HAS-BLED Score for Prediction of Bleeding and Mortality After Transcatheter Aortic Valve Replacement

**DOI:** 10.21470/1678-9741-2021-0331

**Published:** 2023

**Authors:** Monirah A. Albabtain, Amr A. Arafat, Haneen Alghasoon, Wiam Abdelsalam, Abdulrahman Almoghairi, Mohammad Alotaiby

**Affiliations:** 1 Cardiology Clinical Pharmacy Department, Prince Sultan Cardiac Center, Riyadh, Saudi Arabia.; 2 Adult Cardiac Surgery Department, Prince Sultan Cardiac Center, Riyadh, Saudi Arabia.; 3 Cardiothoracic Surgery Department, Tanta University, Tanta, Egypt.; 4 Cardiac Research Department, Prince Sultan Cardiac Center, Riyadh, Saudi Arabia.; 5 Adult Cardiology Department, Prince Sultan Cardiac Center, Riyadh, Saudi Arabia.; 6 Adult Cardiology Department, Saud Al-Babtain Cardiac Center, Dammam, Saudi Arabia.

**Keywords:** Transcatheter Aortic Valve Replacement, Bleeding, Mortality, Body Surface Area, Hospital Mortality

## Abstract

**Introduction:**

Bleeding after transcatheter aortic valve replacement (TAVR) is associated with increased mortality. The predictive value of the HAS-BLED score in TAVR patients is still to be evaluated. We assessed the value of the HAS-BLED score to predict in-hospital bleeding and mortality after TAVR and the impact of diferent renal impairment definitions on the predictive value of the score system.

**Methods:**

We retrospectively included 574 patients who underwent TAVR at a single center. Study outcomes were 30-day mortality and the composite endpoint of major and life-threatening bleeding as defined by The Valve Academic Research Consortium-2. The predictive value of the HAS-BLED score was calculated and compared to a modified model. The performance of the score was compared using two definitions of renal impairment. Model discrimination was tested using C-statistic and the Net Reclassification Index.

**Results:**

Bleeding occurred in 78 patients (13.59%). HAS-BLED category 3 was a significant predictor of bleeding (OR: 1.99 ]1.18- 3.37], C-index: 0.56, *P*=0.01). C-index increased to 0.64 after adding body surface area and extracardiac arteriopathy to the model. The Net Reclassification Index showed an increase in the predic tive value of the model by 11.4% (*P*=0.002). The C-index increased to 0.61 using renal impairment definition based on creatinine clearance. Operative mortality was significantly associated with the HAS-BLED score (OR: 7.54 [95% CI: 2.73- 20.82], C-index: 0.73, *P*<0.001).

**Conclusion:**

The HAS-BLED score could be a good predictor of in-hospital mortality after TAVR. Its predictive value for bleeding was poor but improved by adding procedure-specific factors and using creatinine clearance to define renal impairment.

## INTRODUCTION

Indications for transcatheter aortic valve replacement (TAVR) are increasingly expanding. The risk of bleeding after TAVR is high due to the age of the patients, previous cardiac surgeries, and several concomitant comorbidities^[1]^. Moreover, bleeding after TAVR is associated with increased mortality^[2,3]^.

The HAS-BLED score is a simple, well-established, clinical bleeding risk prediction score for 1-year bleeding in patients with atrial fibrillation (AF)^[4]^. Several studies demonstrated the clinical efficacy of the HAS-BLED score for bleeding risk stratification with superior performance compared with other specific scores for bleeding or other cardiovascular events and long-term outcomes^[5,6]^.

The predictive value of the HAS-BLED score has been studied in patients with acute coronary syndrome receiving dual or triple antithrombotic therapy and showed moderate accuracy^[7,8,9]^. There is increasing interest in adopting a score that predicts bleeding for patients undergoing a cardiac procedure such as TAVR. This study aimed to evaluate the HAS-BLED score as a predictive tool for 30-day mortality and bleeding following TAVR. Additionally, we assessed the impact of changing the definition of renal impairment on the predictive value of the HAS-BLED score.

## METHODS

### Design and Patients

This retrospective study included patients who underwent TAVR at a single center between April 2009 and July 2020. We excluded patients who underwent transapical or transaortic TAVR, patients with an aborted procedure, or those who required emergency cardiac surgery. Additionally, we excluded patients lacking any of the HAS-BLED components needed to calculate the score. A total of 574 patients were included in our analysis.

### Data and Definitions

Baseline clinical characteristics, medical history, laboratory data, treatments administered, and 30-day occurrence of adverse events were collected. The HAS-BLED score was calculated based on clinical and laboratory data at admission. Data were retrieved from our prospectively maintained TAVR registry. We used the Valve Academic Research Consortium-2 (VARC-2) definition of postoperative bleeding^[10]^. The HAS-BLED score consists of several components; each component was given one point, and the risk of bleeding was directly proportional to the HAS-BLED score^[11]^. The risk of bleeding was divided into three categories based on the HAS-BLED score; low risk had a score of zero, moderate risk had a score of 1-2, and high risk had a HAS-BLED score ≥3^[12,13]^. We grouped categories one and two into one category.

Uncontrolled hypertension was defined as systolic blood pressure of 160 mmHg or higher. Liver impairment was considered when bilirubin was higher than twice the normal level; liver enzymes were higher than three times the normal values or the presence of cirrhosis. We used the renal impairment definition proposed in the original HAS-BLED score (dialysis, transplant, creatinine >2.26 mg/dL or >200 µmol/L). Moreover, we used the Cockcroft-Gault creatinine clearance calculator [creatinine clearance (mL/min) = (140-age (years)) × weight (kg) × (0.85 if female)/[72 × serum creatinine (mg/dL)]. We tested the change in the predictive value of the score using the definition based on creatinine clearance (<50 mL/min) or dialysis.

### Study Outcomes

Our endpoint was 30-day mortality and the composite of major and life-threatening bleeding according to the VARC-2 definitions.

### Ethical Consideration

The study was approved by the Institutional Review Board of the hospital (Reference number: R21003), and the need for patient consent was waived. This study was performed in compliance with the Declaration of Helsinki (7^th^ version released in 2013).

### Statistical Analysis

Continuous data were presented as mean and standard deviation, and categorical data were presented as frequencies and percentages. Univariable logistic regression analysis was performed to identify factors predicting the composite endpoint of major and life-threatening bleeding. Variables with a *P*<0.1 were included in the multivariable regression model with HAS-BLED. Backward elimination was performed to keep variables with a *P*<0.1 in the final multivariable regression model. The calibration of the models was tested using the Hosmer-Lemeshow test and model discrimination was tested using C-statistic to report the area under the curve (AUC). The Net Reclassification Index was used to test the change in the predictive value of the new models compared to the original HAS-BLED score. Stata 16.1 (StataCorp, College Station, TX, USA) was used to perform the statistical analysis.

## RESULTS

### Preoperative Data

A total of 574 patients were included in the study. Demographic and preoperative characteristics are presented in Table 1. The mean age was 76.2±9 years, 60.28% were male, and severe renal impairment was diagnosed in 5.57% of the patients. Patients over 65 years represented 86.41% of our cohort, and 508 (88.50%) patients were using antithrombotics and anticoagulants.

**Table 1:** Patients’ perioperative characteristics.

Variable	N=574
Age, years	76.16±9.04
Male, n (%)	346 (60.28%)
BMI, kg/m^2^	30.67±6.53
BSA, m^2^	1.82±0.21
EuroSCORE II, %	4.7±6.45
Uncontrolled hypertension, n (%)	85 (14.81%)
Diabetes, n (%)	366 (63.76%)
Renal impairment, n (%)	
Severe	32 (5.57%)
Dialysis	21 (3.66%)
Extracardiac arteriopathy, n (%)	52 (9.06%)
Previous stroke, n (%)	49 (8.54%)
Liver impairment, n (%)	8 (1.39%)
Chronic lung disease, n (%)	86 (14.98%)
Previous AVR, n (%)	13 (2.26%)
Previous mitral valve surgery, n (%)	9 (1.57%)
Previous CABG, n (%)	52 (9.06%)
Prior bleeding	10 (1.74%)
NYHA class III/IV, n (%)	464 (80.84%)
AF, n (%)	74 (12.89%)
Recent MI, n (%)	25 (4.36%)
Previous PCI	141 (24.56%)
Previous PPM	15 (2.61%)
**Laboratory data**	
Hemoglobin, mg/dL	12.35±1.89
Platelets	253.16±85.18
Albumin	37.69±4.22
Creatinine, µmol	104.64±85.13
Creatinine clearance, mL/min	67.13±30.35
Bilirubin	10.04±20.21
Troponin T	0.11±0.53
**Medications**	
Warfarin, n (%)	23 (4.01%)
P2Y12, n (%)	275 (47.91%)
ASA	451 (78.57%)
NOAC	48 (8.36%)

AF=atrial fibrillation; ASA=acetylsalicylic acid; AVR=aortic valve replacement; BMI=body mass index; BSA=body surface area; CABG=coronary artery bypass grafting; CCS=Canadian Cardiovascular Society; MI=myocardial infarction; NOAC=non-vitamin K oral anticoagulants; NYHA=New York Association; PCI=percutaneous coronary intervention; PPM=permanent pacemaker

Uncontrolled hypertension was reported in 14.81% of the patients, previous stroke in 8.54%, liver impairment in 1.39%, and previous bleeding in 1.74%. The HAS-BLED scores were divided into two categories: 454 patients (79.09%) had low or moderate risk of bleeding, and 120 (20.91%) had a high risk of bleeding.

### HAS-BLED and Postoperative Bleeding

**Fig. 1:**
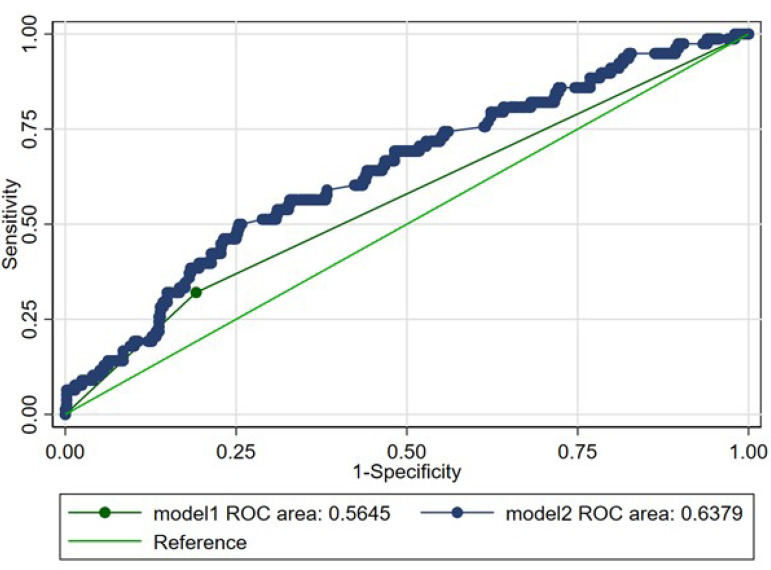
Receiver operating characteristic curve for bleeding and HAS-BLED score (model 1) and HAS-BLED score, body surface area, and extracardiac arteriopathy (model 2).

Major and life-threatening bleeding was reported in 78 patients (13.59%). Univariable risk factors for bleeding were female gender (*P*=0.047), body surface area (BSA) (*P*=0.002), extracardiac arteriopathy (*P*=0.04) and HAS-BLED score ≥3 (*P*=0.01). Multivariable analysis for factors affecting bleeding is presented in Table 2. The C-index of the HAS-BLED score increased from 0.56 (95% confidence interval [CI]: 0.51-0.61) to 0.64 (95% CI: 0.57-0.70) (*P*=0.02) after adding BSA and extracardiac arteriopathy (Figure 1).

**Table 2:** Multivariable logistic regression for factors affecting bleeding.

Bleeding	OR (95% CI)	*P*-value
BSA	0.15 (0.05-0.51)	0.002
Extracardiac arteriopathy	1.84 (0.91-3.86)	0.09
HAS-BLED score ≥3	1.8 (1.07-3.16)	0.03

BSA=body surface area

The Net Reclassification Index was used to test the change in the new model's predictive value after adding BSA and extracardiac arteriopathy compared to the original score. There was an increase in the predictive value of the model by 4.2% (*P*=0.04) with a 6% risk of bleeding and 11.4% (*P*=0.002) with an 8% risk of bleeding.

### Renal Impairment Definitions and HAS-BLED Score

**Fig. 2:**
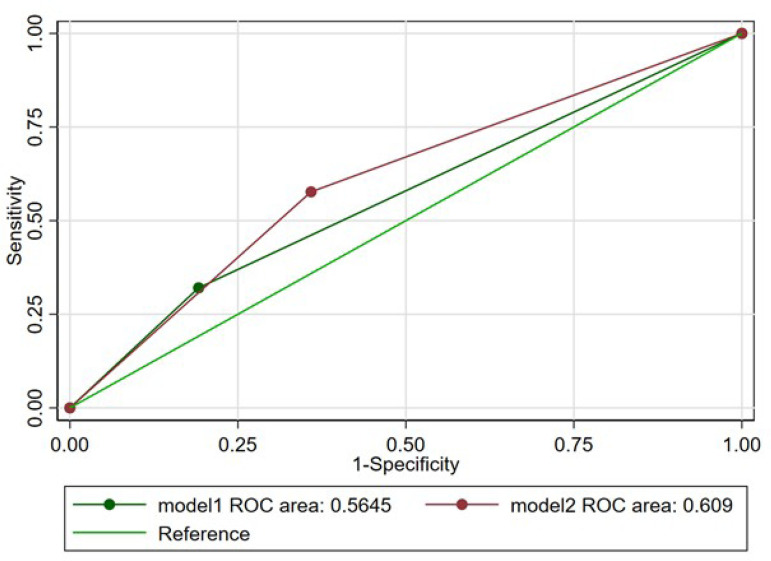
Receiver operating characteristic curve for bleeding and HAS-BLED categories with the original renal impairment definition (model 1) and the renal impairment definition based on creatinine clearance (model 2).

The predictive value of HAS-BLED was retested using the creatinine clearance <50 mL/min as the renal impairment definition (based on EuroSCORE II definition). The C-index increased to 0.61 (95% CI: 0.55-0.67) with the new definition of renal impairment (*P*=0.09) (Figure 2). The Net Reclassification Index was used to test the change in the predictive value of the HAS-BLED score using both definitions of renal impairment. Renal impairment was added to the other HAS-BLED score components, and the change in the predictive value with each definition was calculated. With a 9% risk of bleeding, there was a 0.2% improvement in the HAS-BLED score when renal impairment was added using the original definition (*P*=0.84), and the improvement was 3.8% with the definition of creatinine clearance (*P*=0.07).

### Association Between HAS-BLED and Mortality

**Fig. 3:**
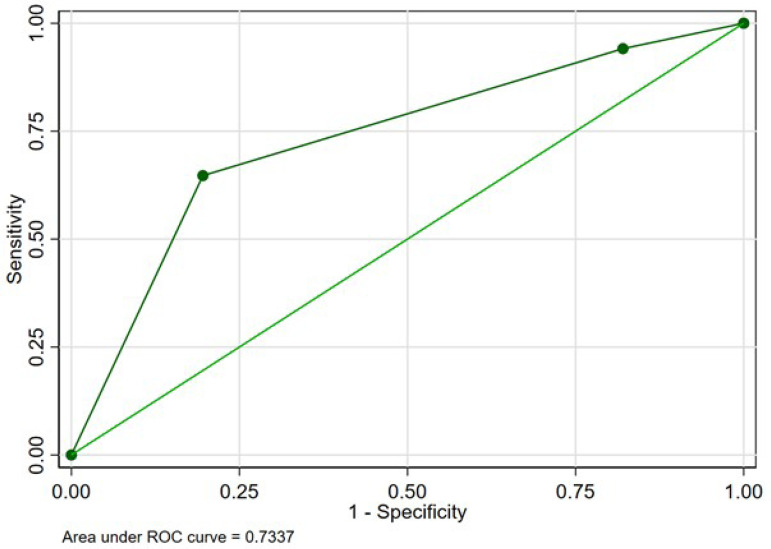
Receiver operating characteristic curve for the association between HAS-BLED score and mortality.

Thirty-day mortality occurred in 17 patients (2.96%). Operative mortality was significantly associated with category 3 of the HAS-BLED score (OR: 7.54 [95% CI: 2.73-20.82], *P*<0.001), and the C-index was 0.73 (Figure 3).

## DISCUSSION

The HAS-BLED score was established to predict 1-year bleeding in patients with AF on anticoagulant therapy. Because of its simplicity and usefulness, this score becomes more prevalent in everyday clinical practice when deciding on starting oral anticoagulants for patients with AF and newly diagnosed acute coronary syndrome who need to be on antiplatelet therapy. Studies on its utility to predict bleeding after TAVR are limited. Patients who undergo TAVR have a high-risk profile and are more labile to several complications such as bleeding. Despite being a simple procedure compared to open surgery, there is a substantial risk of bleeding after TAVR.

Risk prediction is crucial in those patients to optimize their therapy preoperatively. We found that HAS-BLED score may have a predictive value for in-hospital bleeding after TAVR. Additionally, we found the predictive value of the HAS-BLED score was increased by adding other factors not included in the original score. Furthermore, we found that the HAS-BLED score is a good predictor of in-hospital mortality.

Transfemoral TAVR showed favorable 30-day and 1-year mortality than surgical aortic valve replacement in patients with aortic stenosis^[14]^. Bleeding risk stratification is becoming mandatory for patients scheduled for TAVR. In a study on 967 patients by Honda et al., the score was a predictor for 1-year severe bleeding and death after TAVR^[15]^. They reported an AUC of 0.71 for bleeding and 0.72 for mortality for a HAS-BLED score of 4 (high HAS-BLED score). In our study, a high HAS-BLED score (≥3) was a significant predictor of major and life-threatening bleeding after TAVR.

One of the major differences between our study and others is that we only used the HAS-BLED score to predict hospital outcomes. We believe that TAVR patients are diferent from AF patients, and the pattern of antithrombotic prescription in those patients is completely diferent. Therefore, it is difficult to predict their 1-year risk of bleeding, and it is more convenient to develop a score to predict operative bleeding. Another study had evaluated the predictive value of the HAS-BLED score for in-hospital outcomes after TAVR with comparable results. Veulemans et al. found that the AUC of the HAS-BLED score for 30-day mortality after transfemoral TAVR was 0.58 and it improved to 0.60 when combining the HAS-BLED with CHA_2_DS_2_-VASC score, while the AUC for major vascular and bleeding events remained the same with both scores (C-index=0.56)^[16]^. They also reported a marked improvement in the predictive value of the score in patients with AF compared to non-AF patients.

Currently, there is no risk score for predicting bleeding after TAVR. We studied the effect of other factors in our sample that may affect bleeding risk; we found female gender, low BSA, and extracardiac arteriopathy as predictors of bleeding after TAVR. These factors could increase the risk of bleeding after TAVR because of the nature of the vascular procedure. Lower BSA indicates small-sized vessels with a higher risk of bleeding, and extracardiac arteriopathy could increase the risk of bleeding during and after vessel manipulation. Other factors such as AF and concomitant antithrombotic therapy did not affect bleeding in our cohort. When we added significant variables to the HAS-BLED score, the predictive value of the model markedly improved. This improvement indicated that other factors could affect bleeding after TAVR than the HAS-BLED score items. Larger studies are recommended for better estimation of the predictive value of HAS-BLED in TAVR.

Renal impairment was reported in 5.6% of our patients. Renal impairment was defined in the original HAS-BLED score based on serum creatinine levels. We tested if the predictive value of the score can change if we use a definition based on creatinine clearance. We found an increase in the C-index when using creatinine clearance (from 0.56 to 0.61). At the same time, we tested the change in the predictive value of the HAS-BLED score by adding the two diferent definitions of renal impairment to the other components of the score, and we found an improvement in the predictive value of the score by 0.2% with the original definition and 3.8% with the creatinine clearance definition. This result is similar to a finding by Suzuki et al. They conducted a study in patients with AF on anticoagulation therapy to evaluate the effect of diferent definitions of renal impairment^[17]^. The study showed that modifying the HAS-BLED score by changing the definition of renal impairment to creatinine clearance improved the predictive value of the HAS-BLED score. The small sample size limits this study. The effect of diferent renal impairment definitions needs further investigation as more data are accumulating from observational studies and reports indicating that not only severe but also moderate renal impairment could be a risk factor to increase bleeding in these populations^[18,19]^.

Similar to Honda et al., we found that HAS-BLED predicted mortality even more accurately than predicting bleeding after TAV R ^[15]^. We demonstrated that category 3 of the HAS-BLED score was a significant predictor of 30-day mortality. This result confirms the findings of a previous study^[15]^, indicating the correlation between a high HAS-BLED score and risk of death. On the other hand, another study found that the predictive value for mortality of EuroSCORE II or STS score was higher than that of the HAS-BLED score after TAVR^[16]^.

The HAS-BLED score includes several modifable risk factors, and preoperative correction of these factors may positively affect outcomes after TAVR. Additionally, this study highlighted the need for a new and improved score to predict bleeding after TAVR considering other procedure-specific risk factors, such as arteriopathy.

### Limitations of the Study

The present study has several limitations. We included a single-center experience, so a relatively small sample size for risk scoring. Our sample included a wide variety of patient characteristics. Additionally, the study is limited by its retrospective design.

## CONCLUSION

The HAS-BLED score could be a good predictor of in-hospital mortality after TAVR. Its predictive value for bleeding is poor but could be further improved by adding specific-procedure factors. The predictive value of HAS-BLED was improved when using creatinine clearance to define renal impairment.
